# Current Non-surgical Management of Knee Osteoarthritis

**DOI:** 10.7759/cureus.40966

**Published:** 2023-06-26

**Authors:** Jessica Amelinda Mintarjo, Eka Poerwanto, Eric Hartono Tedyanto

**Affiliations:** 1 Physical Medicine and Rehabilitation, Hang Tuah University, Surabaya, IDN; 2 Neurology, Universitas Udayana/Sanglah General Hospital, Denpasar, IDN

**Keywords:** interventional pain management, regenerative procedures, regenerative medicine therapies, osteoarthritis, knee osteoarthritis/ koa

## Abstract

Osteoarthritis (OA) is a common chronic degenerative disease. The prevalence tends to increase with age and is influenced by underlying risk factors such as gender, obesity, joint injuries (work/sports activities), and geographic region. OA has a distinctive picture, namely, damage to the joint cartilage and the formation of new bone at the edges of the bones, also called osteophytes, due to biochemical, metabolic, physiological, and pathological changes in the joint cartilage and subchondral bone. Symptoms that can be caused include joint pain, inhibition of joint movement, crepitus, deformity, asymmetrical swelling of the joints, signs of inflammation, and changes in gait. Currently, there are various methods of managing OA in terms of reducing pain, including regeneration and non-regeneration therapy. Non-regeneration treatments include physiotherapy (exercise, biomechanical intervention, electrotherapy, diathermy), pharmacology, intra-articular injections (corticosteroids, hyaluronic acid, geniculate nerve blocks), extra-articular injections, and radiofrequency. In comparison, regeneration management includes laser and intra-articular injection (prolotherapy and PRP).

## Introduction and background

Osteoarthritis (OA) is a common chronic degenerative disease. This disease causes damage to joint tissue, including articular cartilage, subchondral bone, and synovium, causing pain and decreased function of the affected joint. Primary OA and secondary OA are the two divisions of OA. The most frequent condition, primary OA, is identified when linked risk factors are present, but there has been no trauma or precipitating illness. Secondary OA develops as a result of an underlying joint condition. Trauma, congenital joint problems, inflammatory arthritis, avascular necrosis, Paget's disease, osteopetrosis, osteochondritis dissecans, hemoglobinopathies, Ehlers-Danlos syndrome, and Marfan's syndrome are among the predisposing conditions. The knee is a frequent site for OA [1-3]. The prevalence of knee osteoarthritis (KOA) in men over 60 years is 5% - 15%, while in women aged 60 years and over, it is 10% - 25%. Risk factors for KOA include age, gender, obesity, joint injuries (work/sports activities), and geographic region. KOA can cause joint pain, muscle weakness, physical disability, and decreased quality of life. In contrast, chronic pain can cause anxiety, depression, and cognitive dysfunction, impacting daily life socially and economically [2-4]. 

KOA is a progressive and multifactorial joint disease. Nearly half of the cases of OA are KOA, and its prevalence increases with age and degree of obesity. Until now, KOA cannot be cured unless knee arthroplasty is performed which is an effective therapy for KOA. However, this is certainly responsible for expensive medical or surgical costs. In some cases, KOA can be managed other than surgery. Pain management and KOA development prevention are the two main objectives of therapy. Regenerative and non-regenerative treatments are the two types that can be administered [3].

## Review

Epidemiology

KOA is the most commonly diagnosed type of arthritis, and its prevalence increases with age and obesity. It is estimated that around 13% of women and 10% of men aged 60 years and over have symptoms of KOA worldwide. The prevalence increases to around 40% for those aged over 70 years. Asymptomatic KOA is estimated at 240 cases per 100,000 persons per year [5,6]. In Indonesia, the prevalence of KOA is still quite high, namely, 15.5% in men and 12.7% in women of Indonesia, which is 255 million people [6].

Etiology

Classification of knee OA is divided into primary and secondary, depending on the cause. Primary knee OA results from articular cartilage degeneration, the exact cause of which is still unknown, presumably due to increasing age. Secondary knee OA results from articular cartilage degeneration with known causes, including post-traumatic, congenital, or limb malformations, malposition (varus/valgus), scoliosis, rachitis, Wilson's disease, gout, pseudogout, rheumatoid arthritis, hemophilia, and Paget's disease [6].

Pathophysiology

Both healthy articular cartilage and healthy articular cartilage maintain a balance between their constituent parts, so any cartilage breakdown is countered by synthesis. Matrix metalloproteases (MMPs), or degradative enzymes, are overexpressed throughout the OA process, disturbing the equilibrium and causing a general decrease in the breakdown of collagen and proteoglycans. The healing procedure is insufficient, resulting in an imbalance that reduces the number of proteoglycans while increasing synthesis, water content, and collagen, finally leading to a loss of articular cartilage flexibility. These modifications cause the cartilage to deteriorate and crack, eroding the articular surface [6].

OA can result from joint inflammation. Cytokines and growth factors are activated during inflammation, which causes the production of degradative enzymes and mediates cartilage degradation. Patients with OA had higher levels of pro-inflammatory cytokines such as interleukin (IL)-1, IL-6, IL-15, IL-17, IL-18, tumor necrosis factor (TNF), and leukemia inhibitory factor (LIF). Interferon (IFN), IL-6, IL-10, IL-4, and TGF- work as anti-inflammatories. Cartilage injury interacts with Toll-like receptors (TLRs), found on the surface of immune cells, to activate the innate immune system, resulting in a sterile inflammatory response. In the synovial tissue, articular bone lesions, and synovial membranes of OA patients, TLR-2 and TLR-4 are elevated. As a result, nitric oxide, prostaglandin E2, and MMPs are increased. Pro-inflammatory cytokines, proteolytic enzymes, and chemokines rise when an inflammatory response is present, whereas anti-inflammatory cytokines and growth factors fall. Joint tissue abnormalities are brought on by this, which is akin to a "vicious circle". Macrophages will discharge pro-inflammatory cytokines that promote CD4+ T cells, angiogenesis, and elevated COX-2 levels in the OA synovium and enhance vascular permeability. Th1-type T cells, in particular, can trigger innate and adaptive immunological responses, inflame synovium, and encourage cartilage degradation. In addition to boosting inflammation and generating autoantibodies specific to chondrocyte surface proteins like collagen and osteopontin, T cells also stimulate B cells [7,8].

Therapy

Currently, there are various methods of managing OA in terms of reducing pain, including regeneration and non-regeneration therapy. Non-regeneration treatments include physiotherapy (exercise, biomechanical intervention, electrotherapy, diathermy), pharmacology, intra-articular (IA) injections (corticosteroids, hyaluronic acid, geniculate nerve blocks), extra-articular injections, and radiofrequency. In comparison, regeneration management includes laser and IA injections (prolotherapy and PRP) [7].

Non-regenerative Medicine

Exercise: Non-pharmacological treatments include 1) avoiding activities that overload the joints, 2) exercising to increase strength, 3) losing weight, and 4) occupational therapy using supports, splints, canes, or crutches [4]. Weight loss is important for overweight and obese patients; every pound of weight loss can reduce the load on the knees 3 to 6 times while also improving physical function and biomechanics in combination with exercise (aerobics or swimming), strength training, and stretching. This will certainly improve the quality of life [1]. Physical activity reduces the severity and increases muscle strength and walking ability in patients with knee OA. Quadriceps strengthening exercises using the quadriceps bench will increase the number of sarcomeres and muscle fibers (actin and myosin filaments needed in muscle contraction), causing an increase in muscle strength. If the exercise is performed repeatedly, it will increase stability and reduce subchondral inflammation in the capsule, thereby reducing pain [9,10].

Biomechanical interventions: Biomechanical interventions modify the onset, progression, and symptoms of OA. A vagus knee brace has shown significant clinical benefits in one year. Various tools that can be used include footwear, foot orthoses, insoles, bracing, and taping. These therapies can readily be employed as supplements to the currently advised core exercise-focused programs, and they include gait aids like canes as well as bracing, taping, orthotics, and footwear. Systematic studies imply that some mechanical therapies (such as knee braces) may reduce pain, while other mechanical interventions or outcomes have produced inconsistent or inconclusive findings [8,11]. 

Electrotherapy: Acute and chronic post-traumatic pain is reduced, as are muscular spasms, muscle stimulation, muscle atrophy, blood circulation, range-of-motion maintenance and improvement, and muscle spasms. These effects and indications can be attained by utilizing electrotherapy (ET). Clinical recommendations promote ET as a short-term, reasonably priced, non-invasive therapy option. It has been shown that electrical stimulation of the quadriceps muscle might lessen weakness and exacerbate knee OA symptoms. Currently used electrical stimulation techniques include pulsed electrical stimulation (PES), non-invasive interactive neurostimulation (NIN), interference current (IFC), transcutaneous high-frequency electrical stimulation (h-TENS), low-frequency transcutaneous electrical stimulation (l-TENS), and neuromuscular electrical stimulation (NMES). By stimulating motor neurons on the skin around the injured knee with H-TENS, movement is made possible by inhibiting mechanoreceptors. Although studies have demonstrated that h-TENS can raise motor arousal and lower voluntary muscle activity, it was previously employed to treat sensory discomfort. The stimulation uses IFC, widely regarded as the "gold standard" for treating knee OA, to transmit currents into the deeper layers of the skin while reducing skin resistance. When employing IFC, patients might improve by as much as 88%, compared to 74% with h-TENS [12-14].

Diathermy: Diathermy is a heat therapy that is widely used as a musculoskeletal therapy. The mechanism is heat transfer to the underlying tissue so that the tissue will experience vasodilation, increase cellular activity, pain threshold, and soft tissue extensibility, and reduce muscle stiffness. Diathermy consists of two forms often used: short-wave diathermy (SWD) and microwave diathermy (MD). SWD uses high-frequency electromagnetic energy to generate heat in certain tissues in pulsed or continuous waves. MD uses microwaves to generate heat in the superficial tissues. Because the waves are of low frequency, they cannot penetrate the deep muscles. The method of action of MD helps provide nutrients and oxygen to the area, increasing local blood flow to hasten tissue healing. In patients with OA of the knee, deep microwave diathermy has been proven to decrease synovial thickness, a predictive sign of missing cartilage, as well as to help lessen the discomfort brought on by synovitis and the loss of cartilage. Deep microwave diathermy at 434 MHz for 30 minutes five times a week is an effective treatment [13,15].

Pharmacotherapy: Non-steroidal anti-inflammatory drugs (NSAIDs) are a group, which have similar therapeutic activities and side effects. The use of NSAIDs in OA patients can be given orally, but some risks can arise, namely, cardiovascular, gastrointestinal bleeding, and kidneys. Because of these side effects, NSAIDs are also recommended as topical therapy as pain relievers. For example, ketoprofen and diclofenac can be used by patients with knee OA [1,11]. COX-I and COX-II were inhibited by non-specific Cox inhibitors (diclofenac, ibuprofen, aspirin, and meloxicam) with minor selectivity in reducing prostaglandin synthesis. Selective Cox-II inhibitors (rofecoxib, celecoxib, and valdecoxib) have fewer side effects in the gastrointestinal tract and efficiently reduce OA pain. Topical therapy can also be given, such as a gel containing diclofenac which can be useful for reducing pain, is easy to use, and has fewer side effects [1,12].

**Figure 1:**
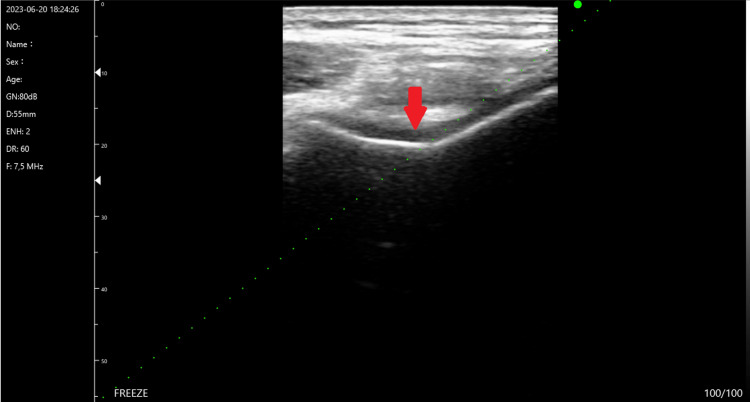
An illustration of ultrasound-guided knee injection (red arrow=suprapatellar bursae; multiple green dots=needle insertion) Image credits: Author

IA corticosteroid injection: IA corticosteroid injections are often conditionally advised in KOA above other IA injections. The injection technique can be blinded or guided by ultrasound. The ultrasound injection site injects steroids into the suprapatellar recess or bursae. An illustration of an ultrasound-guided knee injection can be seen in Figure 1. There is proof that glucocorticoid injections are far more effective than other medications. Corticoids exert their immunosuppressive and anti-inflammatory effects by acting directly on nuclear receptors, disrupting the inflammatory cascade. They decrease the activity and generation of interleukin-1, prostaglandin, leukotriene, MMP9, and MMP-11, which are thought to promote joint mobility in KOA and are a component of the process of pain alleviation. With rising quantities of hyaluronic acid (HA), the anti-inflammatory benefits of this action include reduced erythema, heat, edema, and inflammatory joint discomfort, as well as enhanced relative viscosity. Injecting a corticosteroid into a muscle lowers acute pain episodes and increases joint mobility, especially when joint inflammation and effusion occur [16].

Several corticosteroids, including methylprednisolone acetate, triamcinolone acetate, triamcinolone hexacetonide, betamethasone acetate, betamethasone sodium phosphate, and dexamethasone, are now approved by the Food and Drug Administration for IA treatment. The most popular ones are triamcinolone acetonide and methylprednisolone acetate. Their average dosage is 40 mg, with injections spaced at least three months apart. In vitro, bacterial fermentation and the production of hyaluronic acid (HA), a viscoelastic mucopolysaccharide component of synovial fluid, are two ways it might be made. N-acetyl glucosamine and glucuronic acid make up the glycosaminoglycan known as HA. The advantages of HA injections are restricted to trials with a higher risk of bias than saline injections, according to the 2019 ACR/European League Against Rheumatism research. Therefore, HA injection has been conditionally advised for reducing joint problems when glucocorticoids or other treatments have not been successful [16].

Studies contrasting corticosteroids with HA are many. HA is more effective in decreasing pain than corticosteroids. Compared to corticosteroids, which last only 2-3 weeks, it takes 3-5 weeks to start having an anti-pain impact and is sustained for 5-13 weeks. HA is a glycosaminoglycan present in all parts of the human body and is crucial for forming articular cartilage and synovial fluid. HA will be harmed in OA patients, leading to pain and stiffness. A joint can produce more HA naturally by being injected with HA, which also serves as a lubricant. Giving PRP had more significant benefits in a different trial than the group who received HA [6,13].

Genicular nerve block (GNB): GNB has just come to light as an alternate method that effectively reduces OA pain and enhances functional status. This is because color Doppler and ultrasonography simplify identifying the geniculate neurons and geniculate arteries [12,17]. The target site of NAM for injection was found to be adjacent to the lateral superior, medial superior, and medial inferior genicular arteries, precisely at the intersection of the epicondyle, femur shaft, and tibia. Doppler ultrasonography was used to validate this location. The suprapatellar bursa, which is referred to as a narrow anechoic space on the mid-longitudinal ultrasound view, deep into the quadriceps tendon and superficial to the femoral metaphysis, serving as a marker to identify the underlying aspect, was the target point for the IACSI (intraarticular corticosteroid injection) group [18].

Injection of the medial collateral ligament: Edema in the medial collateral ligament has been described as a sign of degenerative meniscal tears or medial knee OA. This may justify administering MCL injections in knee OA as PRP may be used to treat chronic injuries [12,17]. The patient is in a supine position with either their legs slightly bent or their hips externally rotated and stretched. It is advised to use the in-plane method. The needle is inserted superior to inferior or inferior to superior to the target ligament once the probe has been positioned longitudinally in the medial femorotibial joint [17].

Pes anserine tendon and bursa: The US injection guide PES, anserine bursa, and tendon accuracy in cadaver experiments was 92%. The hip is rotated externally, the knee is gently bent, and the probe is positioned in a coronal oblique position on the anteromedial inferior side of the knee. The long-axis in-plane approach may be used to carry out the injection [17].

Popliteus tendon sheath: When comparing the longitudinal and transverse techniques for injecting the popliteus tendon sheath, it is concluded that the longitudinal approach could be more precise. It could result in over-injection into the knee joint, though. The patient is positioned with the contralateral leg twisted inward and the knee slightly flexed in the lateral recumbent posture. The proximal end of the probe was placed on the anterosuperior lateral femoral epicondyle and angled. This enables the lateral collateral ligament, which is situated above the popliteus tendon and sulcus, to be seen. The in-plane approach is not advised if nearby structures, such as the peroneal nerve, have been damaged [17,19,20].

Radiofrequency: Patients with significant joint pain who refuse to have a total knee arthroplasty (TKA) have frequently employed a variety of radiofrequency (RF) therapies, such as radiofrequency ablation (RFA), cold radiofrequency ablation (CRF), and pulsed radiofrequency ablation (PRF). RFA causes tissue harm by thermal mediation in generally uniform, distinct lesions. The benefit of RFA is that it can precisely heat small tissue portions (less than 1 mm), which will be in direct contact with the ablation electrode (between 45 and 50 °C) during the procedure. RF therapy can enhance joint performance and reduce discomfort [21-23]. Most research shows that RFA is a safe and efficient treatment for lowering knee pain and enhancing function within 3-12 months. The therapeutic value of RF for chronic pain that advances beyond joint-mediated pain to peripherally innervated sites is increasing with the development of CRF ablation and non-ablative PRF treatment. Based on 1009 patients from 15 RCTs, meta-analysis by Liu et al. revealed that employing RF as a therapy proved efficient after 12 weeks [23]. A four-week follow-up following treatment, however, showed no appreciable change. These findings suggest that RF has a highly effective healing impact on knee OA patients. Although RF can lessen discomfort and enhance functionality, the outcomes might differ. The Kellgren-Lawrence OA criteria (grades 1-4 produce variable reactions), cycles, time, and temperature are some of the causes of this. The presence of local anesthetics, gender, mental health conditions, and diabetes mellitus can all impact how RF works [23].

Regenerative Medicine

Laser: Recently several investigators have focused on the laser's role in managing OA, although this has yet to be clinically recommended. Ahmad researched the effects of low- and high-intensity laser therapy on pain, stiffness and knee function in OA patients [10]. When combined with exercise, both high- and low-level laser therapy was more effective in reducing symptoms than exercise alone or placebo laser. According to another study by Robbins, in the short term, low-level laser therapy (LLLT) combined with stretching exercises gives good pain and tissue repair results. Disability was also significantly reduced at the recommended LLLT dose compared to the placebo, i.e. at moderate levels at the end of therapy [11,12].

IA injection of platelet-rich plasma: Injections of platelet-rich plasma (PRP) are promising as a KOA therapy. PRP has been proven to be a safe and efficient therapy in several randomized studies. IA PRP is not yet a routine treatment for KOA, but in active young individuals with low-grade OA, it is more effective than HA [16]. The newest option that can be used to trigger cartilage regeneration is platelet-rich plasma (PRP). PRP is an autologous algorithm of human platelets consisting of growth factors secreted by platelets to support mesenchymal tissue repair. Thus, it helps in the management of degenerative articular cartilage lesions and OA. PRP has three granular components: α-granules, solid granules, and lysosomal granules. Each granule has its function; α-granules are a source of growth factors such as PDGF (platelet-derived growth factor), IGF-1 (insulin-like growth factor), VEGF (vascular endothelial growth factor), TGF (transforming growth factor) ), PF4 (platelet factor 4), and FGF (fibroblast growth factor); these factors have a role in the process of cell proliferation and differentiation and modulate inflammatory molecules so that PRP can form areas for tissue regeneration. PRP can stimulate the formation of the extracellular matrix and improve metabolic balance to help the tissue healing process [18]. PRP also reduces adipogenesis and inflammation in IFP (Infrapatellar Fat Pad). IFP triggers genu OA by promoting adiponectin and leptin, inducing cartilage degradation and leukocyte and monocyte infiltration in OA. PRP reduces IFP inflammation by downregulating proinflammatory cytokines and adipokines, stimulating cartilage formation [19].

Prolotherapy injection: Prolotherapy is a non-surgical procedure by giving injections to treat chronic pain in musculoskeletal conditions, including knee OA. The injection is given by applying hypertonic dextrose to the ligament and tendon insertions to treat musculoskeletal pain; infiltration is generally mixed using local anesthesia [20]. Dextrose prolotherapy is an alternative and promising injection-based therapy for managing chronic musculoskeletal pain [21]. Dextrose will increase the occurrence of angiogenesis and apoptosis through the VEGF, PDGF, IGF, CASP3, and CASP8 genes, thus triggering the wound-healing process by administering prolotherapy [24]. Prior to the procedure, the patient must stop taking NSAIDs for 2 - 3 days before the procedure so that it is possible to trigger an inflammatory response. Possible side effects include hemarthrosis and post-injection pain [25-27]. Although dextrose prolotherapy is clinically successful, it has not been recognized as the mainstay of knee OA treatment in recent medicine. The Osteoarthritis Research Society International (OARSI) prohibits dextrose prolotherapy due to very low-quality evidence. This is because there are still many studies that need to be more appropriate regarding the effectiveness of this therapy [21,28-31].

## Conclusions

There are various therapeutic modalities for patients with knee osteoarthritis. There is no one treatment option that is superior to other therapies. Each individual has a variety of symptoms and different degrees of severity. Therapy is selected based on indications tailored to each patient's condition. With a variety of therapeutic modalities, it is hoped that patients with knee osteoarthritis can be treated comprehensively to obtain satisfactory results.
